# Bis(1,2,3-benzotriazolium) sulfate dihydrate

**DOI:** 10.1107/S1600536813007472

**Published:** 2013-03-28

**Authors:** Randolph K. Belter, Frank R. Fronczek

**Affiliations:** a Y-Not Chemical Consulting, 14400 Williams Road, Zachary, LA 70791, USA; b Department of Chemistry, Louisiana State University, Baton Rouge, LA 70803, USA

## Abstract

In the asymmetric unit of the title hydrated salt, 2C_6_H_6_N_3_^+^·SO_4_^2−^·2H_2_O, there are two independent sulfate ions, one lying on a twofold axis, and the other in a general position. There are three independent benzotriazolium cations and three independent water mol­ecules. The sulfate ion in a general position forms hydrogen-bonded chains of stoichiometry SO_4_^2−^·3H_2_O in the *b*-axis direction. The sulfate on the twofold axis is unhydrated and accepts hydrogen bonds from four surrounding benzotriazoles. The benzotriazolium cations form two types of stacks along *b*. One stack contains only one type of independent cation, related by inversion centers. The other stack contains two alternating independent cations and no symmetry. The two types of stacks have orientations which are rotated by about 79° in the *ac* plane. 12 symmetrically distinct hydrogen bonds of type N—H⋯O(sulfate), N—H⋯O(water), O—H⋯O(sulfate) and O—H⋯O(water), with donor–acceptor distances in the range 2.5490 (13)–2.7871 (12) Å, form a three-dimensional array.

## Related literature

For the structure of benzotriazole hydrogensulfate, see: Giordano (1980 ▶); Meléndez *et al.* (1996 ▶); Ramos-Organillo & Contreras (2007 ▶). For the structure of benzotriazolium dihydrogen phosphate, see: Emsley *et al.* (1985 ▶) and for the structure of benzotriazolium perchlorate monohydrate, see: Sieroń (2007 ▶). For the preparation and purification of benzotriazole with discussion of impurities, see: Damschroder & Peterson (1955 ▶); Miller & Schlaudecker (1958 ▶); Howard & Popplewell (1967 ▶); Spatz & Evans (1973 ▶). For a purification method for aryl­triazoles as their sulfate salts, see: Belter (2013 ▶).
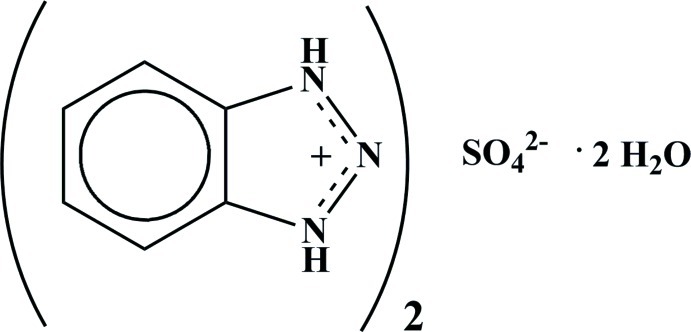


## Experimental

### 

#### Crystal data

2C_6_H_6_N_3_^+^·SO_4_^2−^·2H_2_O
*M* *_r_* = 372.37
Monoclinic, 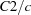

*a* = 38.312 (3) Å
*b* = 6.7621 (10) Å
*c* = 20.987 (2) Å
β = 113.410 (5)°
*V* = 4989.5 (10) Å^3^
*Z* = 12
Mo *K*α radiation
μ = 0.24 mm^−1^
*T* = 90 K
0.28 × 0.22 × 0.18 mm

#### Data collection

Bruker Kappa APEXII DUO CCD diffractometer
Absorption correction: multi-scan (*SADABS*; Sheldrick, 2004 ▶) *T*_min_ = 0.936, *T*_max_ = 0.958
33853 measured reflections
9017 independent reflections
7722 reflections with *I* > 2σ(*I*)
*R*_int_ = 0.023

#### Refinement

*R*[*F* ^2^ > 2σ(*F* ^2^)] = 0.033
*wR*(*F* ^2^) = 0.098
*S* = 1.03
9017 reflections
375 parameters
30 restraints
H atoms treated by a mixture of independent and constrained refinement
Δρ_max_ = 0.56 e Å^−3^
Δρ_min_ = −0.46 e Å^−3^

### 

Data collection: *APEX2* (Bruker, 2006 ▶); cell refinement: *SAINT* (Bruker, 2006 ▶); data reduction: *SAINT*; program(s) used to solve structure: *SHELXS97* (Sheldrick, 2008 ▶); program(s) used to refine structure: *SHELXL97* (Sheldrick, 2008 ▶); molecular graphics: *ORTEP-3 for Windows* (Farrugia, 2012 ▶); software used to prepare material for publication: *SHELXL97*.

## Supplementary Material

Crystal structure: contains datablock(s) I, global. DOI: 10.1107/S1600536813007472/zl2540sup1.cif

Structure factors: contains datablock(s) I. DOI: 10.1107/S1600536813007472/zl2540Isup2.hkl

Supplementary material file. DOI: 10.1107/S1600536813007472/zl2540Isup3.cml

Additional supplementary materials:  crystallographic information; 3D view; checkCIF report

## Figures and Tables

**Table 1:** Hydrogen-bond geometry (Å, °)

*D*—H⋯*A*	*D*—H	H⋯*A*	*D*⋯*A*	*D*—H⋯*A*
N1—H1*N*⋯O3	0.96 (1)	1.65 (1)	2.5981 (11)	173 (1)
N3—H3*N*⋯O2*W* ^i^	0.96 (1)	1.59 (1)	2.5492 (11)	176 (1)
N4—H4*N*⋯O4	0.96 (1)	1.63 (1)	2.5822 (10)	170 (1)
N6—H6*N*⋯O6^ii^	0.94 (1)	1.66 (1)	2.5834 (11)	170 (1)
N7—H7*N*⋯O3*W*	0.94 (1)	1.68 (1)	2.6119 (11)	172 (1)
N9—H9*N*⋯O5	0.96 (1)	1.62 (1)	2.5735 (11)	178 (1)
O1*W*—H11*W*⋯O2^iii^	0.85 (1)	1.98 (1)	2.7498 (10)	151 (2)
O1*W*—H12*W*⋯O2	0.84 (1)	1.96 (1)	2.7871 (11)	172 (2)
O2*W*—H21*W*⋯O1^iv^	0.84 (1)	1.90 (1)	2.7345 (11)	170 (2)
O2*W*—H22*W*⋯O1*W*	0.83 (1)	1.82 (1)	2.6507 (12)	178 (2)
O3*W*—H31*W*⋯O3	0.83 (1)	1.87 (1)	2.7034 (10)	176 (2)
O3*W*—H32*W*⋯O1^iv^	0.81 (1)	1.96 (1)	2.7594 (11)	169 (2)

Symmetry codes: (i) 
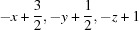
; (ii) 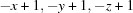; (iii) 
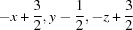
; (iv) 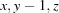.

**Fig. 1.**
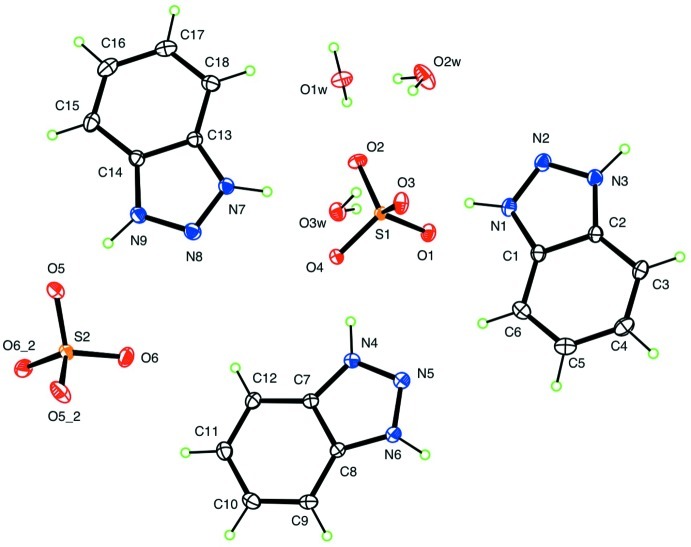
: The asymmetric unit, also including symmetry-related (1 - x, y, 3/2 - z) O atoms on sulfate ion S2. Ellipsoids are drawn at the 50% level.

**Fig. 2.**
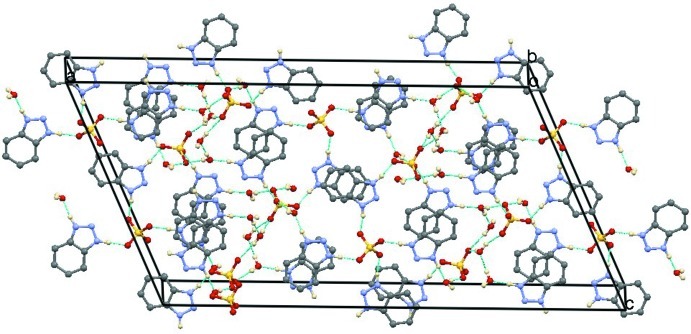
: The unit cell, viewed down the b axis.

## supplementary crystallographic information

### Comment

Benzotriazole is used mainly as a corrosion inhibitor in aqueous based industrial cooling systems. In the preparation of benzotriazole from *o-*benzenediamine, the product benzotriazole is generally heavily contaminated with dark-colored impurities. Mention of such impurities can be found in the preparations and purifications of benzotriazole in Damschroder & Peterson (1955), Miller & Schlaudecker (1958), Howard & Popplewell (1967), and Spatz & Evans (1973). In a recently developed methodology for the purification of crude product aryltriazoles, it has been discovered that the sulfate salt of benzotriazole can be precipitated from acidic aqueous solution as a pure white hydrate, see Belter (2013). To determine the extent of hydration and to validate the stoichiometry of the salt, we crystallized the title benzotriazolium sulfate dihydrate, (1), (C_6_H_6_N_3_^+^)_2_^.^ SO_4_^-2.^ 2H_2_O, from water at ice-bath temperature.

Upon melting point determination (flat stage), the crystals were observed to go through several transitions before melting. Crystals become opaque at >35°C, clarified at 64°C and softened at 72°C before melting at 114–116°C.

The crystal structures of two polymorphs of benzotriazole hydrogensulfate have been reported (Giordano, 1980; Meléndez *et al.*, 1996; Ramos-Organillo & Contreras, 2007). The structures of benzotriazolium dihydrogen phosphate (Emsley *et al.*, 1985) and benzotriazolium perchlorate monohydrate (Sieroń; 2007) have also been reported. In the above literature, we observe that for unhydrated benzotriazolium salts (HSO_4_^-^ and H_2_PO_4_^2-^), hydrogen bonded chains of the anions dictate the packing, with hydrogen-bonded cations bridging between chains.

For a hydrated benzotriazolium salt (HClO_4_^-.^ H_2_O) the packing is dictated by the clustering of anion-water units of 2 ClO_4_^-.^ 2 H_2_O, which are further hydrogen bonded into columns. The cations crosslink the columns by hydrogen bonds, to water on one side, and to perchlorate on the other.

Our compound, 1, as a hydrate, displays similar clustering of anions and water. In this case, sulfates S1 are found as clusters containing 2 SO_4_^2-^ and 6 H_2_O, which are stacked to form infinite columns in the b direction. "Anhydrous" sulfates S2 lie on twofold axes and are isolated from other sulfates and water molecules, accepting hydrogen bonds only from cations.

In the current structure, the benzotriazolium cations bridge between anions and anion-water columns, as seen in the hydrogensulfate and perchlorate structures, having elements of both hydrated and anyhydrous types. Cation N1, N2, N3 bridges between S1 sulfate and water, cation N4, N5, N6 bridges between S1 and S2 sulfates, and cation N7, N8, N9 bridges water and S2 sulfate. All told, there are 12 hydrogen bonds, of which four are *N*—*H*···*O*(sulfate), two are *N*—*H*···*O*(water), five are *O*—*H*(water)···sulfate, and one is *O*—*H*(water)···water.

The benzotriazolium cations form two types of stacks in the b direction. One stack contains only one type of independent cation, that containing N4, N5, and N6, with cations related by inversion centers. The other stack contains the other two alternating independent cations (N1, N2, N3 and N7, N8, N9) and no symmetry. The two types of stacks have orientations which are rotated by about 79° in the *ac* plane.

### Experimental

36.0 g (0.30 mol) of 98% benzotriazole flakes were stirred vigorously with 73.5 g (0.15 mol) of a hot solution of 20% H_2_SO_4_ in an erlenmeyer flask whereupon the entirety dissolved. The mixture was allowed to cool several hours in an ice-water bath whereupon precipitation had occurred. The precipitate was vacuum filtered to yield a white filter cake which proved to be 11.6% by weight of water, determined by Karl-Fisher titration. A second crop of precipitate was collected as crystals after continued icing of the mother liquor. One of these crystals was selected for X-ray analysis.

### Refinement

H atoms on C were placed in idealized positions with C—H distances 0.95 Å and thereafter treated as riding. Coordinates of the NH and water hydrogen atoms were refined, with all N—H distances restrained to be equal and all O—H distances also restrained to be equal. *U*_iso_ for H were assigned as 1.2 times *U*_eq_ of the attached atoms (1.5 for water).

### Crystal data

**Table:**

2C_6_H_6_N_3_^+^·SO_4_^2^^−^·2H_2_O	*F*(000) = 2328
*M**_r_* = 372.37	*D*_x_ = 1.487 Mg m^−^^3^
Monoclinic, *C*2/*c*	Melting point: 387 K
Hall symbol: -C 2yc	Mo *K*α radiation, λ = 0.71073 Å
*a* = 38.312 (3) Å	Cell parameters from 9903 reflections
*b* = 6.7621 (10) Å	θ = 3.1–32.5°
*c* = 20.987 (2) Å	µ = 0.24 mm^−^^1^
β = 113.410 (5)°	*T* = 90 K
*V* = 4989.5 (10) Å^3^	Needle fragment, colourless
*Z* = 12	0.28 × 0.22 × 0.18 mm

### Data collection

**Table:**

Bruker Kappa APEXII DUO CCD diffractometer	9017 independent reflections
Radiation source: fine-focus sealed tube	7722 reflections with *I* > 2σ(*I*)
TRIUMPH curved graphite monochromator	*R*_int_ = 0.023
φ and ω scans	θ_max_ = 32.6°, θ_min_ = 2.0°
Absorption correction: multi-scan (*SADABS*; Sheldrick, 2004)	*h* = −57→58
*T*_min_ = 0.936, *T*_max_ = 0.958	*k* = −10→8
33853 measured reflections	*l* = −31→30

### Refinement

**Table:**

Refinement on *F*^2^	Primary atom site location: structure-invariant direct methods
Least-squares matrix: full	Secondary atom site location: difference Fourier map
*R*[*F*^2^ > 2σ(*F*^2^)] = 0.033	Hydrogen site location: inferred from neighbouring sites
*wR*(*F*^2^) = 0.098	H atoms treated by a mixture of independent and constrained refinement
*S* = 1.03	*w* = 1/[σ^2^(*F*_o_^2^) + (0.0567*P*)^2^ + 2.9928*P*] where *P* = (*F*_o_^2^ + 2*F*_c_^2^)/3
9017 reflections	(Δ/σ)_max_ = 0.001
375 parameters	Δρ_max_ = 0.56 e Å^−^^3^
30 restraints	Δρ_min_ = −0.46 e Å^−^^3^

### Special details

**Table:**

Geometry. All e.s.d.'s (except the e.s.d. in the dihedral angle between two l.s. planes) are estimated using the full covariance matrix. The cell e.s.d.'s are taken into account individually in the estimation of e.s.d.'s in distances, angles and torsion angles; correlations between e.s.d.'s in cell parameters are only used when they are defined by crystal symmetry. An approximate (isotropic) treatment of cell e.s.d.'s is used for estimating e.s.d.'s involving l.s. planes.
Refinement. Refinement of *F*^2^ against ALL reflections. The weighted *R*-factor *wR* and goodness of fit *S* are based on *F*^2^, conventional *R*-factors *R* are based on *F*, with *F* set to zero for negative *F*^2^. The threshold expression of *F*^2^ > σ(*F*^2^) is used only for calculating *R*-factors(gt) *etc*. and is not relevant to the choice of reflections for refinement. *R*-factors based on *F*^2^ are statistically about twice as large as those based on *F*, and *R*- factors based on ALL data will be even larger.

### Fractional atomic coordinates and isotropic or equivalent isotropic displacement parameters (Å^2^)

**Table:**

	*x*	*y*	*z*	*U*_iso_*/*U*_eq_	
N1	0.67711 (2)	0.46488 (13)	0.46788 (4)	0.01481 (14)	
H1N	0.6750 (4)	0.478 (2)	0.5115 (6)	0.018*	
N2	0.71156 (2)	0.45131 (13)	0.46877 (4)	0.01585 (15)	
N3	0.70747 (2)	0.43361 (12)	0.40372 (4)	0.01438 (14)	
H3N	0.7307 (3)	0.423 (2)	0.3965 (7)	0.017*	
C1	0.64988 (3)	0.45610 (14)	0.40201 (5)	0.01312 (15)	
C2	0.66993 (2)	0.43532 (13)	0.35953 (5)	0.01262 (15)	
C3	0.65153 (3)	0.42098 (14)	0.28708 (5)	0.01530 (16)	
H3	0.6651	0.4051	0.2581	0.018*	
C4	0.61243 (3)	0.43150 (15)	0.26072 (5)	0.01780 (18)	
H4	0.5986	0.4238	0.2119	0.021*	
C5	0.59194 (3)	0.45342 (15)	0.30363 (5)	0.01893 (18)	
H5	0.5650	0.4598	0.2826	0.023*	
C6	0.61000 (3)	0.46569 (15)	0.37485 (5)	0.01706 (17)	
H6	0.5963	0.4797	0.4037	0.020*	
N4	0.56493 (2)	0.74001 (12)	0.53044 (4)	0.01388 (14)	
H4N	0.5909 (3)	0.737 (2)	0.5631 (6)	0.017*	
N5	0.55857 (2)	0.72941 (13)	0.46390 (4)	0.01556 (15)	
N6	0.52140 (2)	0.73277 (13)	0.42951 (4)	0.01439 (14)	
H6N	0.5123 (4)	0.720 (2)	0.3812 (6)	0.017*	
C7	0.53172 (2)	0.74983 (13)	0.54006 (5)	0.01227 (15)	
C8	0.50299 (2)	0.74511 (14)	0.47330 (5)	0.01227 (15)	
C9	0.46433 (3)	0.75356 (14)	0.46170 (5)	0.01505 (16)	
H9	0.4447	0.7508	0.4163	0.018*	
C10	0.45658 (3)	0.76613 (15)	0.52029 (5)	0.01654 (17)	
H10	0.4308	0.7714	0.5151	0.020*	
C11	0.48569 (3)	0.77145 (15)	0.58815 (5)	0.01650 (17)	
H11	0.4788	0.7803	0.6269	0.020*	
C12	0.52368 (3)	0.76409 (14)	0.59952 (5)	0.01481 (16)	
H12	0.5432	0.7684	0.6449	0.018*	
N7	0.62831 (2)	0.09067 (12)	0.71668 (4)	0.01345 (14)	
H7N	0.6316 (4)	0.110 (2)	0.6751 (6)	0.016*	
N8	0.59310 (2)	0.08433 (13)	0.71241 (4)	0.01494 (15)	
N9	0.59523 (2)	0.06704 (12)	0.77634 (4)	0.01462 (14)	
H9N	0.5716 (3)	0.057 (2)	0.7817 (7)	0.018*	
C13	0.65399 (2)	0.07740 (13)	0.78380 (5)	0.01246 (15)	
C14	0.63215 (2)	0.06263 (13)	0.82335 (5)	0.01298 (15)	
C15	0.64890 (3)	0.04931 (15)	0.89610 (5)	0.01695 (17)	
H15	0.6342	0.0411	0.9233	0.020*	
C16	0.68810 (3)	0.04896 (16)	0.92546 (5)	0.01979 (19)	
H16	0.7008	0.0398	0.9745	0.024*	
C17	0.71011 (3)	0.06174 (16)	0.88508 (5)	0.01946 (19)	
H17	0.7371	0.0598	0.9080	0.023*	
C18	0.69386 (3)	0.07690 (15)	0.81376 (5)	0.01660 (17)	
H18	0.7087	0.0864	0.7867	0.020*	
S1	0.667426 (6)	0.69362 (3)	0.616423 (10)	0.01027 (5)	
O1	0.67023 (2)	0.85284 (11)	0.57055 (4)	0.01523 (13)	
O2	0.700031 (19)	0.69702 (11)	0.68344 (4)	0.01676 (14)	
O3	0.66609 (2)	0.50055 (10)	0.58176 (4)	0.01686 (13)	
O4	0.632125 (19)	0.71736 (11)	0.62831 (3)	0.01558 (13)	
S2	0.5000	0.17120 (5)	0.7500	0.01520 (7)	
O5	0.53233 (2)	0.04538 (13)	0.79305 (4)	0.02273 (16)	
O6	0.51148 (2)	0.29683 (12)	0.70401 (4)	0.02015 (15)	
O1W	0.73087 (2)	0.31970 (12)	0.71948 (4)	0.01937 (14)	
H11W	0.7519 (4)	0.323 (2)	0.7544 (7)	0.029*	
H12W	0.7236 (4)	0.4369 (19)	0.7109 (8)	0.029*	
O2W	0.73056 (2)	0.10807 (13)	0.61293 (5)	0.02518 (17)	
H21W	0.7140 (4)	0.019 (2)	0.6024 (9)	0.038*	
H22W	0.7300 (5)	0.174 (2)	0.6458 (8)	0.038*	
O3W	0.63174 (2)	0.16552 (11)	0.59721 (4)	0.01566 (13)	
H31W	0.6424 (4)	0.2700 (19)	0.5942 (8)	0.023*	
H32W	0.6421 (4)	0.079 (2)	0.5841 (8)	0.023*	

### Atomic displacement parameters (Å^2^)

**Table:**

	*U*^11^	*U*^22^	*U*^33^	*U*^12^	*U*^13^	*U*^23^
N1	0.0177 (3)	0.0159 (4)	0.0116 (3)	−0.0010 (3)	0.0067 (3)	−0.0015 (3)
N2	0.0166 (3)	0.0175 (4)	0.0129 (3)	−0.0016 (3)	0.0052 (3)	−0.0011 (3)
N3	0.0143 (3)	0.0165 (4)	0.0127 (3)	−0.0016 (3)	0.0058 (3)	−0.0015 (3)
C1	0.0152 (4)	0.0124 (4)	0.0127 (4)	−0.0003 (3)	0.0066 (3)	−0.0011 (3)
C2	0.0143 (3)	0.0123 (4)	0.0119 (3)	−0.0012 (3)	0.0060 (3)	−0.0009 (3)
C3	0.0203 (4)	0.0142 (4)	0.0113 (4)	−0.0023 (3)	0.0060 (3)	−0.0012 (3)
C4	0.0199 (4)	0.0154 (4)	0.0139 (4)	−0.0018 (3)	0.0023 (3)	−0.0009 (3)
C5	0.0151 (4)	0.0167 (4)	0.0216 (4)	0.0004 (3)	0.0038 (3)	−0.0006 (3)
C6	0.0154 (4)	0.0167 (4)	0.0206 (4)	0.0007 (3)	0.0088 (3)	−0.0010 (3)
N4	0.0120 (3)	0.0156 (4)	0.0133 (3)	0.0006 (3)	0.0043 (3)	0.0002 (3)
N5	0.0150 (3)	0.0170 (4)	0.0146 (3)	0.0007 (3)	0.0059 (3)	0.0004 (3)
N6	0.0144 (3)	0.0162 (4)	0.0121 (3)	0.0008 (3)	0.0048 (3)	0.0004 (3)
C7	0.0122 (3)	0.0110 (4)	0.0128 (4)	0.0002 (3)	0.0041 (3)	−0.0001 (3)
C8	0.0129 (3)	0.0116 (4)	0.0115 (3)	0.0004 (3)	0.0040 (3)	0.0005 (3)
C9	0.0116 (3)	0.0144 (4)	0.0166 (4)	0.0003 (3)	0.0028 (3)	0.0011 (3)
C10	0.0137 (4)	0.0151 (4)	0.0217 (4)	0.0013 (3)	0.0079 (3)	0.0014 (3)
C11	0.0187 (4)	0.0156 (4)	0.0176 (4)	0.0009 (3)	0.0097 (3)	0.0003 (3)
C12	0.0166 (4)	0.0151 (4)	0.0122 (4)	0.0002 (3)	0.0051 (3)	−0.0003 (3)
N7	0.0130 (3)	0.0153 (3)	0.0124 (3)	−0.0001 (3)	0.0054 (3)	−0.0010 (3)
N8	0.0131 (3)	0.0168 (4)	0.0147 (3)	−0.0005 (3)	0.0054 (3)	−0.0018 (3)
N9	0.0136 (3)	0.0164 (4)	0.0150 (3)	0.0004 (3)	0.0069 (3)	−0.0006 (3)
C13	0.0128 (3)	0.0119 (4)	0.0128 (4)	0.0004 (3)	0.0051 (3)	−0.0006 (3)
C14	0.0140 (3)	0.0124 (4)	0.0131 (4)	0.0008 (3)	0.0061 (3)	−0.0007 (3)
C15	0.0233 (4)	0.0153 (4)	0.0134 (4)	0.0022 (3)	0.0086 (3)	0.0006 (3)
C16	0.0242 (4)	0.0175 (4)	0.0135 (4)	0.0027 (4)	0.0030 (3)	0.0011 (3)
C17	0.0151 (4)	0.0194 (4)	0.0190 (4)	0.0013 (3)	0.0015 (3)	0.0011 (3)
C18	0.0132 (4)	0.0180 (4)	0.0180 (4)	0.0007 (3)	0.0056 (3)	0.0003 (3)
S1	0.01024 (9)	0.01164 (10)	0.00885 (9)	−0.00031 (6)	0.00372 (7)	−0.00058 (7)
O1	0.0187 (3)	0.0137 (3)	0.0150 (3)	−0.0005 (2)	0.0085 (2)	0.0023 (2)
O2	0.0125 (3)	0.0202 (3)	0.0128 (3)	−0.0007 (2)	−0.0001 (2)	−0.0005 (2)
O3	0.0262 (3)	0.0126 (3)	0.0154 (3)	−0.0030 (3)	0.0120 (3)	−0.0030 (2)
O4	0.0120 (3)	0.0237 (4)	0.0123 (3)	0.0019 (2)	0.0062 (2)	0.0018 (2)
S2	0.01142 (12)	0.02288 (16)	0.01203 (13)	0.000	0.00544 (10)	0.000
O5	0.0154 (3)	0.0314 (4)	0.0247 (4)	0.0064 (3)	0.0115 (3)	0.0105 (3)
O6	0.0196 (3)	0.0289 (4)	0.0114 (3)	−0.0045 (3)	0.0055 (3)	0.0018 (3)
O1W	0.0163 (3)	0.0192 (3)	0.0177 (3)	0.0024 (3)	0.0016 (3)	0.0022 (3)
O2W	0.0202 (3)	0.0281 (4)	0.0335 (4)	−0.0091 (3)	0.0172 (3)	−0.0119 (3)
O3W	0.0173 (3)	0.0141 (3)	0.0177 (3)	−0.0001 (2)	0.0092 (3)	0.0004 (2)

### Geometric parameters (Å, º)

**Table:**

N1—N2	1.3159 (11)	C12—H12	0.9500
N1—C1	1.3626 (12)	N7—N8	1.3167 (11)
N1—H1N	0.955 (11)	N7—C13	1.3634 (11)
N2—N3	1.3162 (11)	N7—H7N	0.940 (11)
N3—C2	1.3671 (11)	N8—N9	1.3167 (11)
N3—H3N	0.963 (11)	N9—C14	1.3661 (11)
C1—C2	1.3959 (12)	N9—H9N	0.956 (11)
C1—C6	1.4044 (13)	C13—C14	1.3969 (12)
C2—C3	1.4023 (12)	C13—C18	1.4017 (12)
C3—C4	1.3772 (13)	C14—C15	1.4044 (13)
C3—H3	0.9500	C15—C16	1.3781 (14)
C4—C5	1.4183 (15)	C15—H15	0.9500
C4—H4	0.9500	C16—C17	1.4158 (15)
C5—C6	1.3775 (14)	C16—H16	0.9500
C5—H5	0.9500	C17—C18	1.3777 (14)
C6—H6	0.9500	C17—H17	0.9500
N4—N5	1.3207 (11)	C18—H18	0.9500
N4—C7	1.3668 (11)	S1—O2	1.4639 (7)
N4—H4N	0.959 (11)	S1—O1	1.4765 (7)
N5—N6	1.3163 (11)	S1—O4	1.4783 (7)
N6—C8	1.3652 (12)	S1—O3	1.4855 (7)
N6—H6N	0.935 (11)	S2—O6^i^	1.4779 (8)
C7—C8	1.3965 (12)	S2—O6	1.4779 (8)
C7—C12	1.4024 (13)	S2—O5	1.4783 (8)
C8—C9	1.4036 (12)	S2—O5^i^	1.4783 (8)
C9—C10	1.3775 (14)	O1W—H11W	0.846 (12)
C9—H9	0.9500	O1W—H12W	0.835 (12)
C10—C11	1.4181 (14)	O2W—H21W	0.841 (13)
C10—H10	0.9500	O2W—H22W	0.830 (13)
C11—C12	1.3792 (13)	O3W—H31W	0.830 (12)
C11—H11	0.9500	O3W—H32W	0.812 (12)
			
N2—N1—C1	111.75 (8)	C12—C11—H11	119.1
N2—N1—H1N	117.3 (8)	C10—C11—H11	119.1
C1—N1—H1N	130.9 (8)	C11—C12—C7	115.99 (8)
N1—N2—N3	106.63 (7)	C11—C12—H12	122.0
N2—N3—C2	111.26 (8)	C7—C12—H12	122.0
N2—N3—H3N	115.7 (8)	N8—N7—C13	111.56 (8)
C2—N3—H3N	133.0 (8)	N8—N7—H7N	117.0 (8)
N1—C1—C2	104.99 (8)	C13—N7—H7N	131.3 (8)
N1—C1—C6	132.90 (9)	N7—N8—N9	106.65 (7)
C2—C1—C6	122.10 (8)	N8—N9—C14	111.41 (8)
N3—C2—C1	105.37 (8)	N8—N9—H9N	116.6 (8)
N3—C2—C3	132.46 (9)	C14—N9—H9N	132.0 (8)
C1—C2—C3	122.17 (8)	N7—C13—C14	105.18 (8)
C4—C3—C2	115.46 (9)	N7—C13—C18	132.38 (9)
C4—C3—H3	122.3	C14—C13—C18	122.44 (8)
C2—C3—H3	122.3	N9—C14—C13	105.20 (8)
C3—C4—C5	122.59 (9)	N9—C14—C15	132.95 (9)
C3—C4—H4	118.7	C13—C14—C15	121.85 (8)
C5—C4—H4	118.7	C16—C15—C14	115.62 (9)
C6—C5—C4	121.94 (9)	C16—C15—H15	122.2
C6—C5—H5	119.0	C14—C15—H15	122.2
C4—C5—H5	119.0	C15—C16—C17	122.32 (9)
C5—C6—C1	115.73 (9)	C15—C16—H16	118.8
C5—C6—H6	122.1	C17—C16—H16	118.8
C1—C6—H6	122.1	C18—C17—C16	122.35 (9)
N5—N4—C7	111.54 (7)	C18—C17—H17	118.8
N5—N4—H4N	117.3 (8)	C16—C17—H17	118.8
C7—N4—H4N	131.2 (8)	C17—C18—C13	115.42 (9)
N6—N5—N4	106.49 (8)	C17—C18—H18	122.3
N5—N6—C8	111.57 (8)	C13—C18—H18	122.3
N5—N6—H6N	116.7 (9)	O2—S1—O1	111.08 (4)
C8—N6—H6N	131.6 (9)	O2—S1—O4	108.95 (4)
N4—C7—C8	105.05 (8)	O1—S1—O4	109.84 (4)
N4—C7—C12	132.93 (8)	O2—S1—O3	109.54 (4)
C8—C7—C12	122.02 (8)	O1—S1—O3	108.59 (4)
N6—C8—C7	105.35 (8)	O4—S1—O3	108.81 (4)
N6—C8—C9	132.65 (8)	O6^i^—S2—O6	109.83 (7)
C7—C8—C9	122.00 (8)	O6^i^—S2—O5	109.04 (4)
C10—C9—C8	115.75 (8)	O6—S2—O5	109.60 (4)
C10—C9—H9	122.1	O6^i^—S2—O5^i^	109.60 (4)
C8—C9—H9	122.1	O6—S2—O5^i^	109.04 (4)
C9—C10—C11	122.39 (8)	O5—S2—O5^i^	109.73 (7)
C9—C10—H10	118.8	H11W—O1W—H12W	106.4 (15)
C11—C10—H10	118.8	H21W—O2W—H22W	109.9 (17)
C12—C11—C10	121.84 (9)	H31W—O3W—H32W	105.2 (15)
			
C1—N1—N2—N3	0.04 (11)	C12—C7—C8—C9	−0.26 (14)
N1—N2—N3—C2	−0.07 (11)	N6—C8—C9—C10	−179.81 (10)
N2—N1—C1—C2	0.01 (11)	C7—C8—C9—C10	−0.20 (13)
N2—N1—C1—C6	−179.36 (10)	C8—C9—C10—C11	0.36 (14)
N2—N3—C2—C1	0.07 (11)	C9—C10—C11—C12	−0.07 (15)
N2—N3—C2—C3	−179.97 (10)	C10—C11—C12—C7	−0.38 (14)
N1—C1—C2—N3	−0.05 (10)	N4—C7—C12—C11	179.76 (10)
C6—C1—C2—N3	179.41 (9)	C8—C7—C12—C11	0.54 (14)
N1—C1—C2—C3	179.99 (9)	C13—N7—N8—N9	0.04 (10)
C6—C1—C2—C3	−0.56 (14)	N7—N8—N9—C14	0.23 (10)
N3—C2—C3—C4	−179.11 (10)	N8—N7—C13—C14	−0.28 (10)
C1—C2—C3—C4	0.84 (14)	N8—N7—C13—C18	179.76 (10)
C2—C3—C4—C5	−0.58 (14)	N8—N9—C14—C13	−0.40 (10)
C3—C4—C5—C6	0.02 (16)	N8—N9—C14—C15	178.88 (10)
C4—C5—C6—C1	0.31 (15)	N7—C13—C14—N9	0.39 (10)
N1—C1—C6—C5	179.24 (10)	C18—C13—C14—N9	−179.64 (9)
C2—C1—C6—C5	−0.04 (14)	N7—C13—C14—C15	−178.98 (9)
C7—N4—N5—N6	0.19 (11)	C18—C13—C14—C15	0.98 (15)
N4—N5—N6—C8	−0.16 (11)	N9—C14—C15—C16	179.94 (10)
N5—N4—C7—C8	−0.14 (10)	C13—C14—C15—C16	−0.89 (14)
N5—N4—C7—C12	−179.45 (10)	C14—C15—C16—C17	0.17 (15)
N5—N6—C8—C7	0.08 (11)	C15—C16—C17—C18	0.50 (17)
N5—N6—C8—C9	179.73 (10)	C16—C17—C18—C13	−0.44 (15)
N4—C7—C8—N6	0.03 (10)	N7—C13—C18—C17	179.67 (10)
C12—C7—C8—N6	179.44 (9)	C14—C13—C18—C17	−0.29 (14)
N4—C7—C8—C9	−179.67 (9)		

Symmetry code: (i) −*x*+1, *y*, −*z*+3/2.

### Hydrogen-bond geometry (Å, º)

**Table:**

*D*—H···*A*	*D*—H	H···*A*	*D*···*A*	*D*—H···*A*
N1—H1*N*···O3	0.96 (1)	1.65 (1)	2.5981 (11)	173 (1)
N3—H3*N*···O2*W*^ii^	0.96 (1)	1.59 (1)	2.5492 (11)	176 (1)
N4—H4*N*···O4	0.96 (1)	1.63 (1)	2.5822 (10)	170 (1)
N6—H6*N*···O6^iii^	0.94 (1)	1.66 (1)	2.5834 (11)	170 (1)
N7—H7*N*···O3*W*	0.94 (1)	1.68 (1)	2.6119 (11)	172 (1)
N9—H9*N*···O5	0.96 (1)	1.62 (1)	2.5735 (11)	178 (1)
O1*W*—H11*W*···O2^iv^	0.85 (1)	1.98 (1)	2.7498 (10)	151 (2)
O1*W*—H12*W*···O2	0.84 (1)	1.96 (1)	2.7871 (11)	172 (2)
O2*W*—H21*W*···O1^v^	0.84 (1)	1.90 (1)	2.7345 (11)	170 (2)
O2*W*—H22*W*···O1*W*	0.83 (1)	1.82 (1)	2.6507 (12)	178 (2)
O3*W*—H31*W*···O3	0.83 (1)	1.87 (1)	2.7034 (10)	176 (2)
O3*W*—H32*W*···O1^v^	0.81 (1)	1.96 (1)	2.7594 (11)	169 (2)

Symmetry codes: (ii) −*x*+3/2, −*y*+1/2, −*z*+1; (iii) −*x*+1, −*y*+1, −*z*+1; (iv) −*x*+3/2, *y*−1/2, −*z*+3/2; (v) *x*, *y*−1, *z*.
